# Drug, nicotine, and alcohol use among exercisers: Does substance addiction co-occur with exercise addiction?^☆^^☆☆^

**DOI:** 10.1016/j.abrep.2017.12.001

**Published:** 2017-12-09

**Authors:** Attila Szabo, Mark D. Griffiths, Rikke Aarhus Høglid, Zsolt Demetrovics

**Affiliations:** a Institute of Psychology, ELTE Eötvös Loránd University, Budapest, Hungary; b Institute of Health Promotion and Sport Sciences, ELTE Eötvös Loránd University, Budapest, Hungary; c Department of Psychology, Nottingham Trent University, Nottingham, United Kingdom

**Keywords:** Alcohol drinking, Cigarette smoking, Exercise dependence, Illicit substance use, Physical activity, Sport

## Abstract

**Background:**

Scholastic works suggest that those at risk for exercise addiction are also often addicted to illicit drugs, nicotine, and/or alcohol, but empirical evidence is lacking.

**Aims:**

The aim of the present work was to examine the co-occurrence of illicit drug, nicotine, and alcohol use *frequency* (prevalence of users) and *severity* (level of problem in users) among exercisers classified at three levels of risk for exercise addiction: (i) asymptomatic, (ii) symptomatic, and (iii) at-risk.

**Methods:**

A sample of 538 regular exercisers were surveyed via the Qualtrics research platform. They completed the (i) Drug Use Disorder Identification Test, (ii) Fagerström Test for Nicotine Dependence, (iii) Alcohol Use Disorder Identification Test, and (iv) Exercise Addition Inventory.

**Results:**

A large proportion (n = 59; 10.97%) of the sample was found to be at risk for exercise addiction. The proportion of drug and alcohol users among these participants did not differ from the rest of the sample. However, the incidence of nicotine consumption was lowest among them. The severity of problematic substance use did not differ across the groups.

**Conclusions:**

These findings suggest that substance addiction and the risk for exercise addiction are unrelated. In fact, those at risk for exercise addiction exhibited the healthiest profile related to the prevalence of smoking.

## Highlights

• Exercise addiction is unrelated to greater prevalence of alcohol or substance use.
• Problematic alcohol or substance use is not a characteristic of exercise addiction.
• The risk for exercise addiction is related to a lower prevalence of nicotine use.

Exercise addiction is described as a psychological dysfunction in which the exercising individual loses control over the exercise behaviour; acts compulsively, exhibits dependence, and experiences negative life consequences (Szabo, Griffiths, & Demetrovics, 2016). At this time, diagnosed cases of exercise addiction do not exist, because there are no official diagnostic criteria for it. Although often classified as a behavioural addiction (Egorov & Szabo, 2013), the DSM-5 in its subsection of “Non-substance-related disorders” in the category of “Substance-related and Addictive Disorders” only includes “gambling disorder” as a form of behavioural addiction (American Psychiatric Association, 2013). Scholars working in the area of exercise addiction have typically adapted the DSM criteria for substance dependence (Hausenblas and Downs, 2002a, Hausenblas and Downs, 2002b), or use the components model of addictions (Griffiths, 2005) as the theoretical underpinning for their work. The components model of addiction comprises six criteria which are claimed to be present in all substance and behavioural addictions (Griffiths, 2005). The Exercise Addiction Inventory (Terry, Szabo, & Griffiths, 2004), a scale for assessing exercise addiction, was conceptualized on the basis of the components model.

Co-occurrence of addictions is supported by many studies (Cook, 1987) indicating that those who are addicted to one behaviour or substance tend to be addicted to several behaviours or substances at the same time (Di Nicola et al., 2015, Konkolÿ Thege et al., 2016, Sussman et al., 2014). It has been reported that exercise addiction might co-occur with other behavioural addictions, such as compulsive buying (Lejoyeux et al., 2008, Müller et al., 2015, Villella et al., 2011). In two empirical studies (Müller et al., 2015, Villella et al., 2011) an association was made on the basis of statistically significant positive correlations between the two behavioural addictions, which in the former emerged to be stronger in women than in men, but still yielding only about 15% of shared variance, while in the latter its value was low ρ = 0.14. In the study by Villella et al. (2011) examining 2853 young participants (aged 13–20 years) also reported statistically significant correlations between the risk for exercise addiction and internet addiction, pathological gambling, and work addiction. However, the values of these correlations ranged between ρ = 0.21 and ρ = 0.26 indicating a low proportion of shared variance between the measures. Instead of correlations, Lejoyeux et al. (2008) demonstrated that the rate of those affected by compulsive buying was higher among exercisers classified to be at-risk for exercise addiction (63%) than in those who were not at-risk (38%). However, the prevalence of exercise addiction was very high in this study (42%), which sheds doubt on the assessment tool used to diagnose exercise addiction. Further, in a later study, the authors failed to replicate their earlier findings on the relationship between the risk for exercise addiction and compulsive buying (Lejoyeux, Guillot, Chalvin, Petit, & Lequen, 2012).

It is generally agreed by those in the exercise addiction field that up to about 3% of the exercising population may be risk for exercise addiction (Mónok et al., 2012), but the rate can be higher, perhaps because of interpretation issues, in elite athletes (Szabo, Griffiths, de la Vega, Mervó, & Demetrovics, 2015). Hausenblas and Downs (2002a) found no differences in addiction scores for age, gender, or type of exercise. This was also replicated in a recent study (Mayolas-Pi et al., 2017). Furthermore, the prevalence rate does not appear to differ between team and individual exercises (Lichtenstein, Larsen, Christiansen, Støving, & Bredahl, 2014). However, Griffiths et al. (2015) suggest that cultural and gender differences may affect the results of these studies. Furthermore, exercise frequency is strongly associated with the risk for exercise addiction (Terry et al., 2004).

The risk for exercise addiction has also been studied in relation to co-occurrence with substance addictions. A study with undergraduates reported that the risk for exercise addiction was significantly related to drinking alcohol and alcohol-related problems (Martin, Martens, Serrao, & Rocha, 2008). However, the findings were based on statistically significant but rather meaningless correlations accounting for < 5% shared variance between the risk for exercise addictions and alcohol use and related problems. Furthermore, the authors showed that only three out of the eight subscales assessing the risk for exercise addiction were consistently related alcohol use and related problems. Another correlational investigation reported negative findings concerning the association between the risk for exercise addiction and alcohol use disorder (Müller et al., 2015). This finding was also confirmed by Lejoyeux et al. (2008) who reported that there was no difference in the prevalence of alcohol consumption between those at-risk and not at-risk for exercise addiction. This was further confirmed in later research in which, however, the severity of the reliance on alcohol was greater in the former group as compared to the latter (Lejoyeux et al., 2012). This finding is important because it highlights that both the frequency of use (prevalence) and severity (level of problem in users) aspects of substance use need to be evaluated when investigating the co-occurrence of various addictions. With regard to co-occurrence of the risk for exercise addiction and nicotine use, Lejoyeux et al. (2008) found that nicotine dependence did not differ in those at-risk and at no risk for exercise addiction, but the cigarette smokers in the former group smoked less than those in the latter group. In the later study, the authors confirmed their earlier findings. However, the prevalence of users and non-users was not reported.

To the best of these authors' knowledge, the association between the level of risk for exercise addiction and illicit drug use has not been studied to date. However, exercisers who use stimulants for performance enhancement might become hooked on them (Freimuth, Moniz, & Kim, 2011). Based on a comprehensive systematic review, Sussman, Lisha, and Griffiths (2011) reported that 15% of those at-risk for exercise addiction may have co-occurring drug, nicotine, and alcohol addictions. In their review, the authors did not locate any study with a sample size of at least 500 participants that found co-occurrence of the risk for exercise addiction with other addictions. Consequently, they urged further research in this area.

Considering that research investigating the co-occurrence between the risk for exercise addiction and substance addictions (i) is often correlational in nature, (ii) examines limited substances, and (iii) typically examines only one dimension (i.e., prevalence, or severity), as well as the lack of research examining the association between exercise and illicit drug use, the present study was designed to address these gaps in the literature. Therefore, the current work examines both the *frequency* (prevalence) and the *severity* (level of problem in users) of three groups of chemical substances that are potentially addictive (i.e., illicit drugs, nicotine, and alcohol) in a heterogeneous group of regular exercisers grouped a posteriori on the basis of their level of risk for exercise addiction as: (i) asymptomatic, (ii) symptomatic, and (iii) at risk for exercise addiction. Based on the findings from past research and reviews, this cross-sectional study examines Sussman et al.'s (2011) hypothesis that: “… *15% of exercise addicts are also addicted to smoking, alcohol, or illicit drugs*…” (p. 12).

## 1 Method

### 1.1 Participants

The research was conducted with ethical approval obtained from a large university's Research Ethics Committee of the Faculty of Education and Psychology at ELTE Eötvös Loránd University. Participants were recruited from various English social media by targeting groups interested in topics connected to sports, exercise and/or physical activities where interested readers were directed to an online survey ran with the *Qualtrics* software (Qualtrics, 2017). The criteria for participation included: participation in regular sports or exercise, the form of which was specifically named, the participant was aged 18 years or over, and that she or he consented to participation. Within a three-month interval, 538 participants meeting these criteria completed fully the online survey. The age of the participants ranged from 18 to 72 years and the average age was 27.45 years (SD = 8.21). They reported taking part in 42 different types of exercise, with a mean frequency of 3.65 occasions per week (SD = 2.50), for an average of 1.24 h each time (SD = 0.87). There were more female (n = 348; 64.7%) than male (n = 190; 35.3%) participants in the sample and the majority of them participated in individual sports (n = 428; 79.6%). The sample was divided in three groups based on their level of risk for exercise addiction (see Materials section below): (i) “asymptomatic” (n = 39), (ii) “symptomatic” (n = 440), and (iii) “at-risk” (n = 59).

### 1.2 Materials

A demographics questionnaire was used to collect data concerning age, gender, type of sport, frequency of exercise, and duration of exercise. The Exercise Addiction Inventory (EAI; Terry et al., 2004) was used to assess the level of risk for exercise addiction. This scale is based on the components model of addiction (Griffiths, 2005) and assesses six common symptoms of addiction: salience, conflict, mood modification, tolerance, withdrawal symptoms, and relapse on a 5-point Likert scale ranging from: 1 = “strongly disagree” to 5 = “strongly agree” (with total scores of between 6 and 30). Risk levels for exercise addiction are: 6–12 = asymptomatic, 13–23 symptomatic, and 24 or above = at-risk. The scale has good psychometric properties (Terry et al., 2004), and the internal consistency (Cronbach α) in the current sample was acceptable (α = 0.71).

The Drug Use Disorders Identification Test (DUDIT; Berman et al., 2003, Hildebrand and Noteborn, 2015) was used to determine the risk level of drug consumption in those participants who admitted using leisure drugs during the past year, and also identified by its name(s) the drug(s) that they have used. The DUDIT is an 11-item scale. Total scores range between 0 and 44 and the higher scores reflect greater drug-related problems. The cut-off score for drug-related problems is 2 for women and 6 for men, while a score of 25 or above is an index of drug dependence for both genders (Berman et al., 2003). In the current sample the scale's internal consistency was excellent (α = 0.91). Only users who responded “yes” to using drugs within the past year were asked to complete the DUDIT.

The Fagerström Test for Nicotine Dependence (FTND; Heatherton, Kozlowski, Frecker, & Fagerström, 1991) was used to assess levels of nicotine addiction in cigarette smokers and snus users. The scale was only completed by those who answered “yes” to smoking cigarettes or smokeless tobacco (snus) within the past year. The total scores can range from “very low addiction” (0–2) to “very high addiction” (8–10). In the current sample the internal consistency of the scale was acceptable (α = 0.76) for cigarette smokers, but it was weaker for snus users (α = 0.68). Only users who responded “yes” to smoking snus or cigarettes were asked to complete the FTND.

The Alcohol Use Disorders Identification Test (AUDIT; Babor, Higgins-Biddle, Saunders, & Monteiro, 2001) was used to assess problematic drinking among those in the sample who answered “yes” to having consumed alcohol in the past year. The 10 items of the scale are divided into three domains: hazardous alcohol use (1–3), alcohol dependence (4–6), and harmful alcohol use (7–10). Cut-off scores are found to be 8–15 for hazardous drinking and a score above 16 reflects serious alcohol use problems. In the current study the internal consistency of the scale was acceptable (α = 0.75). Only users, who responded “yes” to drinking alcohol were asked complete the AUDIT.

### 1.3 Procedure

Interested participants from various social media, mainly groups sharing interest in sports and exercise, were provided an online link to the *Qualtrics* online research platform (Qualtrics, 2017) having a unique Uniform Resource Locator (URL) for the current study. To access the questions, they had to read a consent form and agree to participation by selecting the “I agree” button. Most participants (n = 522; 97%) completed the study in under 7 min. Data were downloaded from the *Qualtrics* platform in an SPSS (Statistical Package for Social Sciences; Version 22.0) data file, verified by two of the researchers for meeting the criteria for participation, completeness of the answers, and absence of errors (i.e., checking outliers) and subsequently analyzed with the same software.

### 1.4 Statistical analyses

To test the null hypothesis that the frequency of illicit drug, nicotine, and alcohol use was not different in asymptomatic, symptomatic, and those at-risk for exercise addiction, the Fisher-Freeman-Halton exact test (Freeman & Halton, 1951), which is an extension of the Fisher exact test for r x c contingency tables, was adopted. To test the null hypothesis that the level of addiction (severity, or problematic use in users) to the three types of substance did not differ between participants who were asymptomatic, symptomatic, and at-risk for exercise addiction, a Kruskal-Wallis test was used instead of a multivariate analysis of variance (MANOVA), because the assumption of normal distribution was violated in the data for the dependent measures and the observations in four cell sizes out of 18 were below 10. When statistically significant results were obtained with the Kruskal-Wallis test, as well as all other comparisons of two independent groups (i.e., gender), the Mann-Whitney *U* test was used.

## 2 Results

### 2.1 Manipulation check for grouping

Level of exercise addiction grouping factors such as age, frequency of exercise, and duration of reported exercise were examined for control and manipulation check purposes. By using the Kolmogorov-Smirnov and Shapiro-Wilk tests it was found that the assumption of normality was violated in these variables (*p* < 0.001). Therefore, the non-parametric Kruskal-Wallis test was employed the determine whether the three measures differed between the groups. The test showed that while the three groups did not differ statistically significantly in age and the reported average duration of their exercise, they differed in the reported weekly frequency of exercise (H(2) = 21.830, *p* < 0.001) with a mean rank of 177.78 for the asymptomatic group, 251.25 for the symptomatic group, and 317.01 for those in the at-risk for exercise addiction group. Between groups differences were further tested with a Mann-Whitney *U* test using Bonferroni correction of the alpha (*p* = 0.017), which demonstrated that those classified at-risk for exercise addiction (Mdn = 5) were different from both symptomatic (Mdn = 4; Z = 3.317, *p* = 0.001) and asymptomatic groups (Mdn = 2; Z = 3.940, *p* < 0.001) and the symptomatic also differed from the asymptomatic group (Z = 3.154, *p* = 0.002). Thus, this manipulation check reinforced the grouping made on the basis of the scale scores.

### 2.2 Gender differences

Given that there were more females than males in the study, gender differences were tested before principal analysis began. Gender differences in the risk for exercise addiction scores were tested with a Mann-Whitney *U* test, and yielded no statistically significant difference (Z = − 0.860, *p* > 0.05) between men (Mdn = 18, mean rank = 261.72) and women (Mdn = 18, mean rank = 273.75). Gender differences among drug, alcohol, and tobacco users were examined with chi-square (*χ*^2^) tests, and these indicated that the ratio of men and women did not differ among the tobacco users (*χ*^2^(1) = 1.11, *p* = 0.292) and alcohol users (*χ*^*2*^[1] = 0.118, *p* = 0.731). However, there were more males than females that used drugs (*χ*^*2*^[1] = 9.67, *p* = 0.003).

### 2.3 Rate of substance use

The Fisher-Freeman-Halton exact tests of the rate of drug and alcohol use among exercisers at three levels of exercise addiction were not significant (Fisher's Exact Test (FET) = 0.959, *p* = 0.631, and FET = 2.058, *p* = 0.325, respectively). However, the test was significant for the prevalence rate of nicotine use (cigarette or snus) use among the three categories of exercise addiction (FET = 8.835, *p* = 0.011). Bonferroni adjusted post-hoc z-tests indicated that the frequency of nicotine use was the lowest (*p* < 0.05) among participants who were at-risk for exercise addiction (18.6%) in contrast to those who were symptomatic (33.6%) or asymptomatic (46.2%), who did not differ between themselves (*p* > 0.05). The prevalence of users and non-users for all the three substances, are shown in Table 1.

**Table 1:** Rounded percentages (and number) of past year users and non-users of illicit drugs, nicotine, and alcohol (*N* = 538) in three groups of exercisers classified on the basis of their level of risk for exercise addiction (EA).

	Asymptomatic (n = 39)		Symptomatic (n = 440)		At risk for EA (n = 59)	
	Non-user	User	Non-user	User	Non-user	User
Illicit drugs	90% (35)	10% (4)	86% (376)	14% (64)	90% (53)	10% (6)
Nicotine	54% (21)	46% (18)	66% (292)	34% (148)	81% (48)	19% (11)^a^
Alcohol	3% (1)	97% (38)	4% (20)	96% (420)	9% (5)	91% (54)

a Statistically significantly different (p < 0.05) rate in contrast to the asymptomatic and the symptomatic groups.

### 2.4 Level of problematic substance use

Examination of the scale data (i.e., DUDIT, FTND and AUDIT) for normality, by using the Kolmogorov-Smirnov and Shapiro-Wilk tests, yielded statistically significant results in all instances (*p* < 0.001), indicating that the assumption of normality was violated in all three dependent measures. Consequently, the non-parametric Kruskal-Wallis test was used to determine whether *users* in the three groups of risk for exercise addiction differed in the severity or problematic drug, nicotine, and alcohol use. None of the three median tests (see Table 2) were found to be statistically significant (*p* > 0.05). Given that the ratio of male and female drug users was different, the Kruskal-Wallis test was repeated for the three groups of risk for exercise addiction separately for men and women. However, similarly to the combined analyses, the results were not statistically significant. The ratio of whether individual and team exercisers differed in the three groups was also examined to rule out the possible effects of the type of sport. The chi-square (*χ*^2^) test yielded no statistically significant difference (*χ*^2^[4] = 1.54, *p* = 0.820). Further, Spearman's rho correlations between the exercise addiction scores and DUDIT, FTND, and AUDIT scores were not statistically significant (*p* > 0.05) either. Finally, the same correlations were repeated, but instead of exercise addiction scores, exercise volume scores were correlated with the outcome variables. Again no statistically significant differences were found (*p* > 0.05). However, risk scores for exercise addiction were weakly, but significantly correlated (Spearman's rho = 0.215, *p* < 0.001) with the reported exercise volumes (frequency × duration).

**Table 2:** Median values on three measures: the Drug Use Disorders Identification Test (DUDIT; the scores range between 0 and 44), the Fagerström Test for Nicotine Dependence (FTDN; the scores range between 0 and 10) and the Alcohol Use Disorders Identification Test (AUDIT; the scores range between 0 and 40) in three risk groups of exercise addiction. No statistically significant differences were found between the three group in any of the three measures.

	Asymptomatic		Symptomatic		At risk for EA	
	n*	Median	n	Median	n	Median
DUDIT	4	2.50	64	4.00	6	6.00
FTDN	18	3.00	148	4.00	11	5.00
AUDIT	38	8.00	420	7.00	54	6.5

Note: n* reflects the number of those who answered “yes” to consuming drugs, tobacco or alcohol in each group and who completed the respective measures.

## 3 Discussion

Using a larger sample than has previously been employed, the present study failed to support Sussman et al.'s (2011) conjecture that about 15% of individuals at-risk for exercise addiction are also addicted to illicit drugs, nicotine, and alcohol. Instead, the results suggest that exercise addiction is an arguably unique type of behavioural addiction, which may co-occur with other potentially addictive but non-stigmatized behaviours, such as shopping (Lejoyeux et al., 2008, Müller et al., 2015) or work (Villella et al., 2011), which are generally perceived as positive, and socially acceptable, forms of behaviour (Egorov & Szabo, 2013). Those concerned with their social image may have perfectionist tendencies which motivates them to engage in non-stigmatized behaviours. Indeed, a recent literature review presented a strong association between the risk for exercise addiction and perfectionism traits (Bircher, Griffiths, Kasos, Demetrovics, & Szabo, 2017), which appears to be negatively related to alcohol use (Pritchard, Wilson, & Yamnitz, 2007). This association may be one of the reasons for the findings in this cross-sectional study demonstrating no empirical evidence that would point towards the co-occurrence of exercise addiction with the generally more stigmatized substance addictions.

While the present authors are not aware of any published studies that have examined the co-occurrence of exercise addiction and illicit drug use, the findings agree with a number of studies showing that participation in organized sports, athletics, or exercise is related to a lower prevalence of illicit drug consumption in young people (Terry-McElrath and O'Malley, 2011, Terry-McElrath et al., 2011). Indeed, epidemiological research shows that higher levels of physical activities are associated with lower levels of drug use (Lynch, Peterson, Sanchez, Abel, & Smith, 2013). Addictive exercise appears to share a common brain mechanism with substance addictions (Martin & Petry, 2005) which may reduce or eliminate the craving for other substances. This explanation is implied in neurobiological models in which exercise is used as a treatment for drug addictions (Lynch et al., 2013). It is also supported by research evidence from animal studies showing that rats who were exposed to progressively increased intensity treadmill exercise (over an 8-week period) preferred saline to amphetamines (Fontes-Ribeiro, Marques, Pereira, Silva, & Macedo, 2011). Although tentative, extrapolating these results to humans suggests that habitual exercise or physical activity may help preventing drug (i.e., amphetamine) addiction. Therefore, the absence of co-occurrence of drug and exercise addiction, as found in the present study, may not be surprising. However, the question of whether addiction to drugs, alcohol, or tobacco, may shift into exercise addiction in humans remains speculative.

With regard to the findings concerning nicotine use, the results concur (at least in part) with those reported by Lejoyeux et al. (2008), but in that study, the number of the cigarettes smoked was the dependent measure rather than the prevalence of smokers among those at-risk for exercise addiction assessed in the present study. Furthermore, here nicotine addiction was considered in a broader sense by also including smokeless tobacco. However, the few who smoked pipes or cigars may have been missed. It appears that the risk for exercise addiction may involve some health concerns that, according Lejoyeux et al. (2012), could explain why such individuals smoke cigarettes less. This explanation is partially supported by two recent studies. One, studying physical education and sport university students, found lower rates of tobacco, alcohol, and Internet use addiction than that generally reported in the literature (Serban & Simona, 2012). The authors attributed the findings to regular participation in sport and physical exercise. Similarly, Martinsen and Sundgot-Borgen (2014) also demonstrated that students in elite sports-specialized high schools used less alcohol, smoked less snus, as well as less cigarettes, than individuals from non-sports specialized, regular high schools. While exercise addiction was not assessed in these studies, the results support the antagonistic effects of exercise and smoking, because the level of sports involvement was higher in sport-specialized high schools than non-specialized high schools. Indeed, one study has argued that it is not physical activity per se, but the pattern of physical activity in favour of stable-high volume patterns, may exert the best protective effects from smoking (Audrain-McGovern, Rodriguez, Rodgers, Cuevas, & Sass, 2012). Through biophysical and/or biochemical mechanisms, exercise may have an antagonistic effect on smoking as it was demonstrated by reduced craving and withdrawal symptoms after exercise (Taylor, Ussher, & Faulkner, 2007). This could be an alternative explanation for lower rate of smokers within those at-risk for exercise addiction, who also reported the largest weekly frequency of exercise, in our present study.

Neither the prevalence rate of alcohol consumption, nor the alcohol-related problematic behaviour, differed in the three groups in the present study. These findings partially agree with that of Lejoyeux et al., 2008, Lejoyeux et al., 2012 who did not find difference in alcohol consumption between those at-risk and those not at-risk for exercise addiction. However, in their later research, problematic alcohol use was greater in those at-risk for exercise addiction than those not at-risk (Lejoyeux et al., 2012). It should also be mentioned that the assessment of problematic drinking relied on different methods in these studies, which could explain, at least in part, the conflicting results. Lejoyeux et al. employed the CAGE (Cut down, Annoyed, Guilty and Eye-opener; Ewing, 1984) with one week retrospective assessments, whereas the present study adopted the AUDIT, which gauges existing and general drinking habits. Furthermore, the assessment of exercise addiction was different in the two studies. Finally, while Lejoyeux et al. (2012) assessed alcohol consumption, the present study assessed prevalence of users in function of the level of risk for exercise addiction and their alcohol use habits. These differences make it difficult to draw a direct parallel explanation between the results of the two studies.

Exercise addiction is arguably unique among the spectrum of both behavioural and chemical addictions in that it has a prerequisite, which is the physical condition to perform high energy-demanding work and the motivation, or self-drive, to achieve a (delayed) reward by investing substantial time and physical effort, often to the point of masochism (Rendi, Szabo, & Szabó, 2007). Individuals at-risk for exercise addiction usually live healthy lives in which exercise has an important role mainly for its physical and psychological effects which have therapeutic benefits, such as stress relief (Szabo, Griffiths, & Demetrovics, 2013). Based on interactional model proposed by Egorov and Szabo (2013), upon experiencing an increased or a suddenly emerging life-stress, these exercise-accustomed people will increase their doses of exercise to compensate for it. It would be easy to turn to illicit drugs, nicotine, or alcohol (and some may), but in preserving a positive social image and hiding the struggle with stress, using a healthy behaviour like exercise is the most viable and socially acceptable source of escape (Egorov & Szabo, 2013). Consequently, while co-occurring addictions do exist (Di Nicola et al., 2015, Konkolÿ Thege et al., 2016, Sussman et al., 2014), it appears that exercise addiction is unlikely to generally co-occur with substance addictions, which can be substantiated on theoretical grounds as well in addition to the results of the present study.

There are some limitations in this study that should be kept in perspective while interpreting its findings. These include the lack of objective exercise measures, the reliance on a self-selected sample, the lack of inclusion of cigars and pipes in the test for nicotine addiction, and the reliance on a single measure for determining the risk for exercise addiction. Furthermore, it should be also noted that while no differences were found in either gender and type of sport in the risk for exercise addiction, females and individual exercisers outnumbered males and team exercisers in the present study, and those classified as symptomatic also outnumbered the asymptomatic and the at risk groups, but this finding was expected, since very few people score on the lower and the higher end of the adopted scale. Future research should address these potentially confounding variables in their research design and use, if possible, a priori random grouping.

## 4 Conclusions

The present study demonstrated that the level of risk for exercise addiction is not associated with an increased use in illicit drugs, nicotine or alcohol. Those at higher risk for exercise addiction include a lower prevalence rate of smokers than those who are symptomatic or asymptomatic. Therefore, in support of past research, heavy involvement in exercise may be antagonistic to smoking behaviour. The severity, or the level of problematic behaviour, associated with illicit drug use, nicotine use, and alcohol use does not differentiate individuals at-risk for exercise addiction from those who are not at-risk. In the present study, by assessing both the frequency (prevalence) and severity (problematic use) of substance use in exercisers grouped into three levels of risk for exercise addiction, there was no evidence for the co-occurrence of substance addiction tendencies among those at risk of exercise addiction.
